# Role of circulating tumour DNA in predicting complete clinical response and local regrowth after total neoadjuvant therapy for rectal cancer: meta-analysis

**DOI:** 10.1093/bjsopen/zrag142

**Published:** 2026-10-05

**Authors:** Nikhil Lal, Siddharth Shukla, Nicola Hodges, Gina Brown, John T Jenkins

**Affiliations:** Department of Surgery and Cancer, Imperial College London, London, UK; Complex Cancer Clinic, St Mark’s the National Bowel Hospital, London, UK; Department of General Surgery, Royal Berkshire NHS Foundation Trust, Reading, UK; Department of Surgery and Cancer, Imperial College London, London, UK; Complex Cancer Clinic, St Mark’s the National Bowel Hospital, London, UK; Department of Surgery and Cancer, Imperial College London, London, UK; Department of Surgery and Cancer, Imperial College London, London, UK; Complex Cancer Clinic, St Mark’s the National Bowel Hospital, London, UK

**Keywords:** non-operative management, watch-and-wait, organ preservation, molecular residual disease, liquid biopsy

## Abstract

**Background:**

Non-operative management after total neoadjuvant therapy (TNT) has emerged as a potential strategy for patients with locally advanced rectal cancer (LARC) achieving clinical complete response (cCR). Circulating tumour DNA (ctDNA) has been proposed as a systemic biomarker to augment response assessment, but its role in predicting cCR and local regrowth remains uncertain.

**Methods:**

A systematic review and meta-analysis were performed of studies evaluating ctDNA dynamics in patients with LARC undergoing TNT. PubMed, EMBASE, and Web of Science databases were searched from database inception to 31 March 2026. Primary outcomes were the association between post-TNT ctDNA status and cCR and sustained cCR. Pooled odds ratios (ORs) were calculated using a random-effects model.

**Results:**

Six studies comprising 597 patients were included. ctDNA negativity following TNT was associated with higher likelihood of cCR (OR 8.01; 95% confidence interval (c.i.) 1.43 to 44.90; *I*^2^ = 27.0%). The association with sustained cCR was not statistically significant (OR 3.46; 95% c.i. 0.03 to 394.64; *I*^2^ = 59.9%). Across studies, post-TNT ctDNA positivity was associated with inferior disease-free, distant recurrence-free, and progression-free survival. ctDNA demonstrated high specificity and positive predictive value for residual disease, but low sensitivity for the detection of residual local disease and local regrowth, with a substantial proportion of patients with residual tumour remaining ctDNA negative at restaging.

**Conclusion:**

Post-TNT ctDNA positivity may identify patients at increased risk of adverse oncological outcomes. However, ctDNA negativity was not consistently associated with the absence of residual local disease, limiting its utility as an independent determinant of organ preservation eligibility. ctDNA should be interpreted as a complementary tool for systemic risk stratification alongside established anatomical assessment.

## Teaser

Circulating tumour DNA is best interpreted as a complementary marker of systemic risk rather than an independent determinant of organ preservation eligibility, which should continue to rest on multimodal anatomical assessment.

## Introduction

Rectal cancer represents a significant global health burden, with an estimated 730 000 new cases diagnosed annually worldwide. Locally advanced (stage II and III) rectal cancer represents a substantial proportion of newly diagnosed rectal cancers and remains a major indication for total neoadjuvant therapy (TNT)^1^. The historical standard of care for this cohort comprised neoadjuvant chemoradiotherapy (nCRT) followed by total mesorectal excision^2^ and, in select patients, adjuvant chemotherapy^3,4^.

TNT^5^, defined as the integration of systemic chemotherapy before or after nCRT or short-course radiotherapy and delivered in its entirety before surgical consideration, has fundamentally altered this landscape^6,7^. Predominantly indicated for proficient mismatch repair or microsatellite stable tumours of the lower and middle rectum, TNT addresses micrometastatic disease earlier in the treatment course, markedly improves chemotherapy compliance, and nearly doubles rates of pathological complete response (pCR) compared with conventional nCRT alone^8–10^.

This surge in response rates has catalysed the adoption of organ preservation strategies, specifically non-operative management (NOM). The magnitude of this shift is evident when comparing clinical guidelines: although the European Society for Medical Oncology (ESMO) 2017 guidelines^3^ previously classified ‘watch and wait’ as an unproven strategy that should only be implemented within prospective trials, the most recent updates, including the 2024 American Society of Clinical Oncology guidelines^6^ and the 2025 ESMO guidelines^7^, now endorse TNT and NOM as preferred standards of care for patients prioritizing organ preservation’.

However, NOM carries a clinically significant and measurable risk of failure. The landmark OPRA trial^9^ demonstrated organ preservation rates of 54% and 39% in patients receiving consolidation and induction chemotherapy sequencing, respectively, with the majority of local regrowth events concentrated within the first 2 years of surveillance. The consequences of undetected regrowth are substantial: tumours identified at an advanced stage of recurrence may no longer be amenable to salvage surgery with curative intent. The accurate and durable identification of complete clinical response (cCR) is therefore not merely a clinical preference, but an oncological imperative.

The current standard for confirming cCR relies on a triad of high-resolution magnetic resonance imaging (MRI), endoscopy, and digital rectal examination^11^, a framework originally described as a three-tier system based on endoscopic and clinical findings in the OPRA trial^9^ and subsequently expanded in clinical practice to incorporate MRI assessment^12^.

Clinicians frequently struggle to distinguish between residual viable tumour cells and the dense fibrosis, inflammation, or mucin pools (that is, the treatment effect) that occur in response to radiation^13^. Furthermore, the tools used for confirming cCR are inherently local; they provide no insight into the systemic biological landscape or the presence of occult micrometastases, which remain the primary drivers of cancer-specific mortality.

To bridge the gap between anatomical imaging and molecular residual disease, circulating tumour DNA (ctDNA) has emerged as a promising biomarker. ctDNA consists of fragmented cell-free DNA shed into the bloodstream by apoptotic or necrotic tumour cells^14^. Unlike traditional protein biomarkers like carcinoembryonic antigen, which suffer from low sensitivity and specificity, ctDNA carries the specific somatic mutations of the primary tumour, effectively serving as a liquid biopsy^15^.

However, the integration of ctDNA into the NOM pathway is complicated by conflicting evidence. Although some studies suggest that persistent ctDNA positivity is a harbinger of local regrowth and systemic failure^16,17^, others have found inconsistent associations^18^. The NEORECT trial^18^ (2024) observed that although undetectable ctDNA was seen in good responders, the marker alone demonstrated low specificity and did not correlate significantly with pCR, suggesting it may not yet be reliable for definitive surgical omission. There is currently no consensus as to whether ctDNA dynamics can reliably stratify patients for organ preservation or predict the specific risk of local regrowth in the NOM setting.

The aim of this systematic review was to specifically synthesize the evidence regarding ctDNA dynamics as a predictor of cCR and local regrowth within the NOM pathway for locally advanced rectal cancer patients undergoing TNT.

## Methods

### Study design and search strategy

This systematic review and meta-analysis was conducted in accordance with the PRISMA 2020 guidelines^19^ (*Table S1*). The study protocol was prospectively registered with PROSPERO (CRD420261322560) and predefined the research question, eligibility criteria, and analytical framework before data collection.

PubMed (MEDLINE), EMBASE, and Web of Science were searched from database inception to 31 March 2026. The search strategy combined controlled vocabulary, including MeSH and Emtree terms, with free-text keywords relating to ‘circulating tumour DNA’, ‘rectal cancer’, and ‘total neoadjuvant therapy’. The full search strategy is provided in *Table S2*. Reference lists of all included studies and relevant systematic reviews were screened manually to identify additional eligible reports.

### Study selection

Studies were eligible if they enrolled patients with histologically confirmed rectal adenocarcinoma undergoing TNT, reported plasma-based ctDNA measurements including a post-treatment assessment, and evaluated the association between ctDNA status and at least one prespecified outcome. Studies were excluded if they did not report rectal cancer-specific data separately, comprised non-original research (including case reports, editorials, and conference abstracts), did not provide extractable outcome data, reported pCR as the sole outcome without assessment of cCR or local regrowth, or did not report post-TNT ctDNA measurements.

Two reviewers (N.L. and S.S.) independently screened all identified records. Titles and abstracts were assessed for relevance, followed by full-text review of potentially eligible studies. Disagreements were resolved through discussion, with arbitration by a third reviewer (N.H.) where necessary.

### Data extraction and outcome definitions

Extracted variables included study characteristics, patient demographics, tumour stage, treatment regimen, and ctDNA assay methodology. Emphasis was placed on the timing of ctDNA sampling (baseline and clinical restaging after TNT), in addition to assay type (tumour informed *versus* tumour agnostic) and detection thresholds, where reported. Three outcomes of interest were prespecified: cCR, sustained cCR (scCR) and survival outcomes. cCR was defined at post-TNT restaging by multimodal assessment based on OPRA criteria (endoscopy and digital rectal examination)^9^, with adjunctive high-resolution MRI incorporated according to study-specific protocols. scCR was defined as the absence of residual or recurrent disease following TNT, operationalized as either sustained cCR without local regrowth in patients managed without surgery or as pCR in those undergoing surgical resection. Survival outcomes comprised disease-free survival, distant recurrence-free survival, and overall survival.

The terms ‘NOM’ and ‘watch and wait’ are used interchangeably in this review to denote planned deferral of surgery with structured surveillance after cCR, whereas ‘organ preservation’ refers to the broader objective of avoiding total mesorectal excision, which may also be achieved by local excision.

### Quality assessment

Methodological quality was assessed using validated tools appropriate to study design. Studies were assessed using the Risk of Bias in Non-randomized Studies of Interventions (ROBINS-I) framework^20^. Domains assessed included confounding, participant selection, outcome measurement, missing data, and selective reporting. Assessments were performed independently by two reviewers (N.L. and S.S.), with discrepancies resolved through consensus discussion.

The certainty of evidence for the pooled and narratively synthesized outcomes was appraised using the GRADE framework.

### Statistical analysis

For outcomes with sufficiently homogeneous definitions across studies, namely cCR and scCR at post-TNT restaging, pooled meta-analyses were performed. Study-level data were used to construct 2 × 2 contingency tables comparing ctDNA-positive and ctDNA-negative groups. Pooled effect estimates were calculated as odds ratios (ORs)^8^ with their 95% confidence interval (c.i.) using a random-effects model, implemented via the meta package in R version 4.4.0 (R Foundation for Statistical Computing, Vienna, Austria), to account for anticipated clinical and methodological heterogeneity.

Statistical heterogeneity was quantified using the *I^2^* statistic and Cochran's Q test. *I*^2^ values of 25%, 50%, and 75% were considered to represent low, moderate, and high heterogeneity, respectively. Prespecified subgroup analyses were not performed owing to the limited number of eligible studies. Survival endpoints were synthesized narratively, with available effect estimates reported descriptively.

## Results

### Study selection

The systematic search identified 248 potential citations across electronic databases. Following the removal of duplicates and the primary screening of titles and abstracts, 34 full-text articles were evaluated for eligibility (*Fig. 1*). Six studies met the inclusion criteria^12,16,21–24^, all reporting longitudinal ctDNA measurements within TNT protocols for locally advanced rectal cancers. Studies using short-course radiotherapy or nCRT without an induction or consolidation chemotherapy component, or those lacking serial ctDNA sampling across a minimum of two time points, were excluded. The final selection comprised three prospective studies^12,22,23^, including two phase II clinical trials (NO-CUT^12^ and NOMINATE^23^), and three retrospective cohort analyses^16,21,24^.

**Figure 1:**
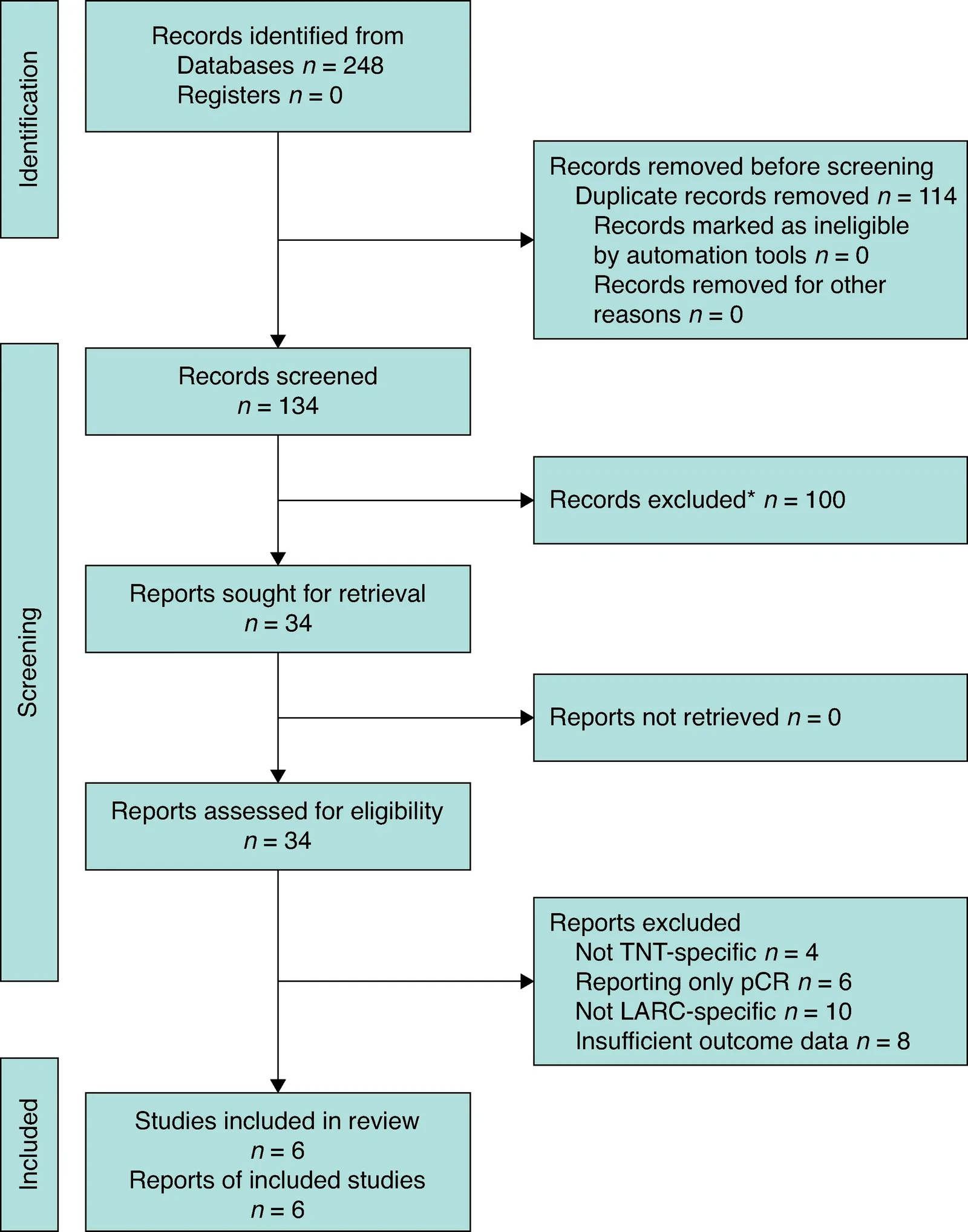
PRISMA flow chart Flow diagram showing the study identification and selection process. In all, 248 records were identified from databases, 114 duplicates were removed, 134 records were screened, 100 were excluded, 34 reports were assessed for eligibility, 28 were excluded with reasons, and 6 studies were included in the review. *Did not meet inclusion criteria.

### Study characteristics

Six studies enrolling 597 patients were included in the final analysis (*Table 1*). The majority of patients presented with T3 or T4 disease, and nodal involvement was identified in 54.7–93.0% of participants across cohorts. Extramural venous invasion (EMVI) positivity was reported in three of six studies^12,22,23^, with rates ranging from 13% to 26%. TNT protocols varied across cohorts, with consolidation chemotherapy sequencing used in three studies^16,21,24^, induction chemotherapy used in one study^12^, and a mixed consolidation and induction approach in two studies^22,23^. Clinical or near-complete response was achieved in 28.6% of patients.

**Table 1:** Characteristics of included studies evaluating ctDNA in locally advanced rectal cancer in patients undergoing TNT

Author (year)	Region	No. of patients	No. of ctDNA samples analysed	T3/4 category	N+	EMVI+	TNT protocol	cCR/nCR
Kim *et al*.^16^ (2025)	USA	28	28	92%	88%		Consolidation	28 (100%)
Amatu *et al*.^12^ (2025)	Italy	179	114	92%	93%	24%	Induction	47 (26%)
Yee *et al*.^21^ (2025)	USA	251	34	80.4%	77.3%		Consolidation	35 (74%)
Manca *et al*.^22^ (2025)	USA	31	31	87%	77%	13%	Consolidation/induction	14 (45.1%)
Alden *et al*.^24^ (2024)	USA	44	44	84%	60%		Consolidation	22 (54%)
Akiyoshi *et al*.^23^ (2025)	Japan	64	64	100%	54.7%	26%	Consolidation/induction	25 (39%)
Total		597	315					171 (28.6%)

Values are *n* (%) unless otherwise stated. A summary is presented of baseline tumour characteristics on magnetic resonance imaging staging, treatment protocols, definitions of cCR, and cCR rates across included cohorts. ctDNA, circulating tumour DNA; TNT, total neoadjuvant therapy; N+, node positive; EMVI, extramural venous invasion; cCR, clinical complete response; nCR, near-complete response.

### ctDNA detection and longitudinal dynamics

Baseline ctDNA positivity was high across all cohorts where reported, ranging from 82% to 98.4%, confirming the utility of ctDNA as a marker of disease burden before treatment. Following completion of TNT, ctDNA positivity declined substantially in all six cohorts, with post-TNT detection rates ranging from 13% to 65%, representing a marked aggregate molecular clearance across the pooled population (*Fig. 2*). The notably higher post-TNT positivity observed in the Manca *et al*.^22^ cohort is likely attributable to the use of an ultrasensitive whole-genome sequencing platform, which carries a lower detection threshold than the tumour-informed assays used across the remaining studies, and should therefore be interpreted in the context of assay methodology rather than reflecting a biological difference in treatment response.

**Figure 2:**
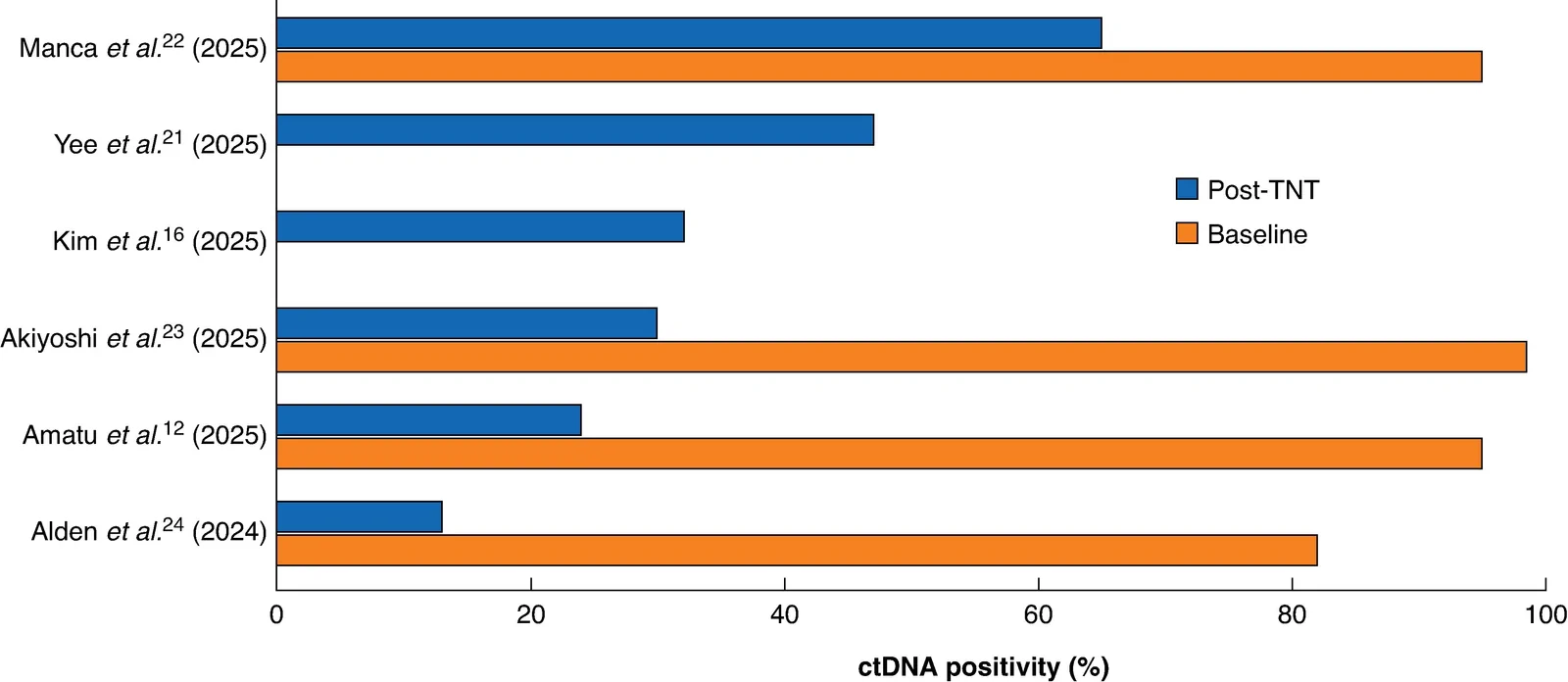
Baseline and post-TNT ctDNA positivity across studies Values represent the proportion of patients with detectable ctDNA at each time point. The chart shows that ctDNA positivity fell from a high baseline (82–98%) to a lower rate after TNT (13–65%) across six studies. Baseline data were not reported for Yee *et al*.^21^ TNT, total neoadjuvant therapy; ctDNA, circulating tumour DNA.

### Heterogeneity in clinical response definitions

Definitions of cCR varied considerably across the included studies (*Table S3*). Although all six cohorts incorporated endoscopy and digital rectal examination as components of response assessment, only three studies explicitly included MRI criteria within their definition of cCR^12,16,24^. Amatu *et al*.^12^ used the modified Memorial Sloan Kettering Cancer Center criteria for response assessment, whereas Manca *et al*.^22^ used the three-tier OPRA trial classification incorporating endoscopy and digital rectal examination.

### ctDNA sampling time points

All six studies obtained baseline plasma ctDNA before commencement of TNT. Sampling during TNT was performed in three cohorts^22–24^, whereas the remaining three studies did not incorporate on-treatment molecular assessment. Post-TNT restaging samples were obtained in four of six studies^12,21,22,24^, with timing varying from approximately 1 week after TNT (Alden *et al*.^24^) to a median of 11–17 weeks^12^, reflecting a lack of standardization in the restaging window across the included literature. Longitudinal surveillance sampling was performed in four cohorts^16,22–24^, although sampling intervals were not consistently reported (*Table S4*).

### ctDNA assay methodology

Four studies used tumour-informed platforms with personalised panels based on patient-specific somatic variants, predominantly via Signatera™ (Natera, Inc.)^16,23,24^, with Manca *et al*.^22^ using whole-genome sequencing via C2i Genomics to quantify tumour fraction rather than binary ctDNA detection. One study^12^ used a tumour-agnostic epigenomic approach via Guardant Reveal and another study^21^ used a mixed strategy incorporating multiple commercial platforms. Detection thresholds were not reported in any included study, precluding direct cross-study comparability of positivity rates (*Table S5*).

### Association between post-TNT ctDNA status and cCR

Four studies^12,21,23,24^ reporting cCR rates in ctDNA-positive and ctDNA-negative patients were included in the pooled analysis. Meta-analysis using a random-effects model demonstrated that ctDNA-negative patients were significantly more likely to achieve cCR than ctDNA-positive patients, with a pooled OR of 8.01 (95% c.i. 1.43 to 44.90; *Fig. 3*). Statistical heterogeneity was moderate across the included studies (*I*^2^ = 27.0%, *P* = 0.250).

**Figure 3:**
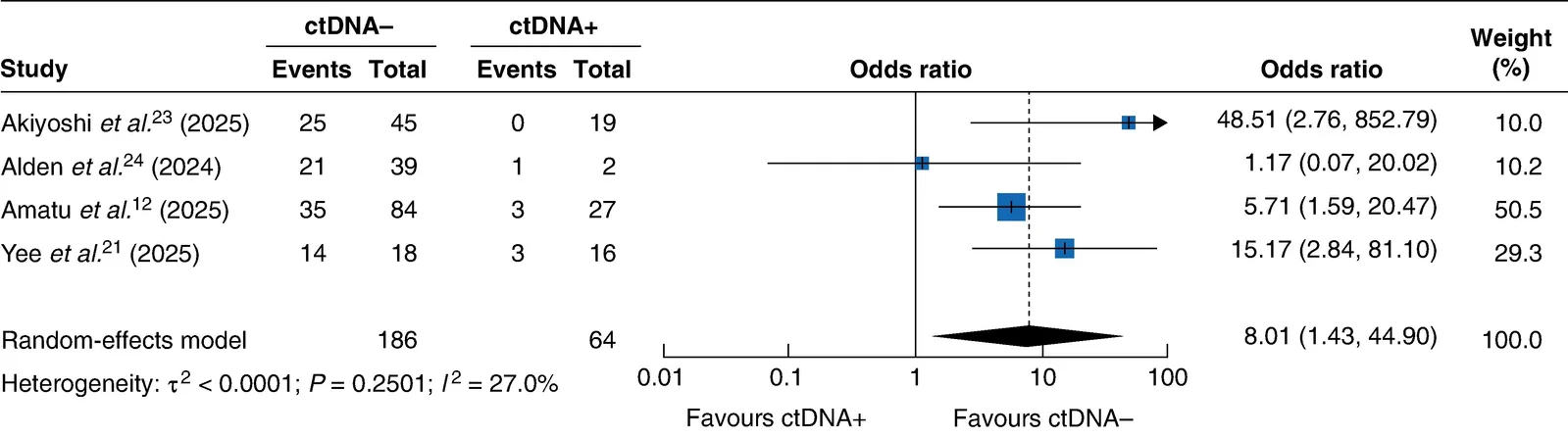
Association between ctDNA status after total neoadjuvant therapy and cCR Forest plot of pooled odds ratios comparing cCR rates in ctDNA-negative *versus* ctDNA-positive patients following total neoadjuvant therapy. Pooled analysis was performed using a random-effects model. Values in parentheses are 95% confidence intervals. Square size is proportional to study weight. The diamond represents the pooled effect estimate with the 95% confidence interval. The forest plot shows that a ctDNA-negative status was associated with higher odds of a cCR. ctDNA, circulating tumour DNA; cCR, clinical complete response.

### Association between post-TNT ctDNA status and scCR

Three studies^16,22,23^ reporting scCR rates in ctDNA-positive and ctDNA-negative patients were included in the pooled analysis. Meta-analysis using a random-effects model produced a pooled OR of 3.46 (95% c.i. 0.03 to 394.64), which did not reach statistical significance (*Fig. 4*). Heterogeneity across the included studies was substantial (*I*^2^ = 59.9%, *P* = 0.083).

**Figure 4:**
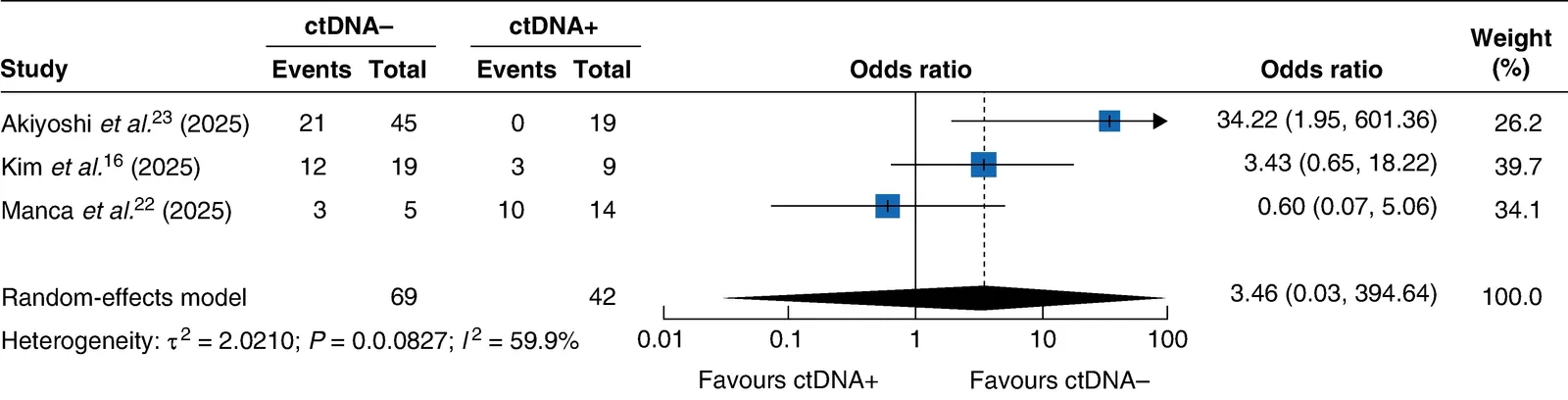
Association between ctDNA status after total neoadjuvant therapy and scCR Forest plot of pooled odds ratios comparing scCR rates in ctDNA-negative *versus* ctDNA-positive patients following total neoadjuvant therapy. Pooled analysis was performed using a random-effects model. Values in parentheses are 95% confidence intervals. Square size is proportional to study weight. The diamond represents the pooled effect estimate with the 95% confidence interval. The forest plot shows a non-significant association between ctDNA-negative status and scCR. ctDNA, circulating tumour DNA; scCR, sustained clinical complete response.

### ctDNA and oncological outcomes

#### Disease-free survival

Akiyoshi *et al*.^23^ reported inferior 18-month disease-free survival in ctDNA-positive patients (hazard ratio (HR) 6.7; 95% c.i. 1.5 to 29.7; *Table 2*). Manca *et al*.^22^ demonstrated that a higher post-TNT tumour fraction was associated with inferior 3-year disease-free survival (HR 4.20; 95% c.i. 1.87 to 9.43).

**Table 2:** Key findings from studies with extractable effect estimates of associations between ctDNA status, oncological outcomes, and diagnostic performance following TNT

Study	Key findings
Akiyoshi *et al*.^23^	Post-TNT ctDNA associated with inferior 18-month DFS (HR 6.7; 95% c.i. 1.5, 29.7) Post-TNT ctDNA specificity 100%, PPV 100% for residual disease Local regrowth occurred in five NOM patients, with ctDNA detected in two during surveillance
Alden *et al*.^24^	Post-TNT ctDNA demonstrated sensitivity (23%), specificity (100%), PPV (100%), and NPV (47%) for detection of residual disease
Amatu *et al*.^12^	Post-TNT ctDNA negativity associated with improved 30-month DRFS (HR 0.38; 95% c.i. 0.17, 0.86) and PFS (HR 0.34; 95% c.i. 0.16, 0.70). Post-TNT ctDNA status not associated with rectal surgery-free survival after NOM (*P* = 0.18)
Manca *et al*.^22^	Higher tumour fraction after TNT associated with reduced scCR (OR 0.10; 95% c.i. 0.01, 0.48) and inferior 3-year DFS (HR 4.20; 95% c.i. 1.87, 9.43)

ctDNA, circulating tumour DNA; TNT, total neoadjuvant therapy; DFS, disease-free survival; HR, hazard ratio; c.i., confidence interval; PPV, positive predictive value; NOM, non-operative management; NPV, negative predictive value; DRFS, distant recurrence-free survival; PFS, progression-free survival; scCR, sustained clinical complete response; OR, odds ratio.

#### Distant recurrence-free and progression-free survival

Amatu *et al*.^12^ reported that ctDNA negativity was associated with improved 30-month distant recurrence-free survival (HR 0.38; 95% c.i. 0.17 to 0.86) and progression-free survival (HR 0.34; 95% c.i. 0.16 to 0.70). In the same cohort^12^, post-TNT ctDNA status was not associated with rectal surgery-free survival in NOM patients (*P* = 0.18).

#### Sustained clinical complete response

Manca *et al*.^22^ demonstrated that a higher post-TNT tumour fraction was associated with reduced scCR (OR 0.10; 95% c.i. 0.01 to 0.48).

#### Diagnostic performance for residual disease and local regrowth

Post-TNT ctDNA exhibited high specificity and positive predictive value for residual disease across reporting cohorts (specificity 100% and positive predictive value 100% in both Akiyoshi *et al*.^23^ and Alden *et al*.^24^).

Sensitivity for residual disease and local regrowth detection was consistently low, with Alden *et al*.^24^ reporting a sensitivity of 23% at restaging and longitudinal surveillance detecting local regrowth in only 40% of patients in the Akiyoshi cohort^23^, indicating that ctDNA negativity does not reliably exclude residual or recurrent local disease (*Table 2*).

### Risk of bias

Methodological quality was assessed using the ROBINS-I framework (*Table S6*). Two studies^12,23^ were rated as at low overall risk of bias, three^16,21,24^ were rated as at moderate risk of bias, and one^22^ was rated as at low to moderate risk of bias. Confounding was the predominant domain of concern across cohorts, reflecting the observational design of the majority of included studies.

Under the GRADE framework, certainty was rated low for the association between ctDNA negativity and cCR and for the association between post-TNT ctDNA positivity and survival, and very low for sustained cCR and for diagnostic accuracy (*Table S7*).

## Discussion

This systematic review and meta-analysis provides the most comprehensive synthesis to date of ctDNA dynamics in patients with locally advanced rectal cancer undergoing TNT and NOM. The central finding is a discordance between molecular and anatomical response: although post-TNT ctDNA positivity identifies patients at elevated risk of incomplete response and adverse oncological outcomes, molecular clearance does not reliably confirm the absence of residual local disease. This distinction reframes the utility of ctDNA in the NOM pathway as a marker of systemic risk stratification rather than a determinant of organ preservation eligibility. These findings address three distinct questions that should not be conflated: prediction of cCR at restaging, detection of local regrowth during surveillance, and prediction of distant recurrence, across which ctDNA performs differently.

Although previous studies^17,25^ have demonstrated an association between ctDNA and pCR in surgical cohorts, this review highlights the distinct challenges of predicting cCR. The pooled association between ctDNA negativity and cCR, although statistically significant, was characterized by wide confidence intervals and moderate heterogeneity, indicating that this relationship is neither consistent nor sufficiently precise to support independent clinical decision-making. The scCR analysis reinforced this uncertainty, with a non-significant pooled estimate and substantial heterogeneity reflecting discordant individual study effects. Due to heterogeneity in definitions of scCR across studies, this analysis should be interpreted as exploratory.

Among composite non-complete response patients in the Akiyoshi *et al*.^23^ cohort, 55% were ctDNA negative at final restaging despite harbouring pathological residual tumour, and 58% of the ctDNA-negative patients who proceeded directly to surgery were found to have pathological (p) T2 or pT3 disease. Following intensive TNT, residual tumour clones sequestered within post-radiation fibrosis or mucinous pools have limited vascular access, reducing DNA shedding into the systemic circulation and generating a substantial false-negative rate that renders ctDNA an unreliable independent gatekeeper for surgical omission^13^. This finding is particularly notable when contextualized against the diagnostic performance of established anatomical modalities: MRI has a sensitivity of 62% for the detection of residual disease and endoscopy has a specificity of 95%, indicating that anatomical assessment retains a discriminatory capability for local disease that current ctDNA platforms do not replicate^24^. ctDNA negativity should not currently be conflated with pathological sterilization of the tumour bed, and decisions regarding organ preservation must continue to be guided by the multimodal anatomical assessment triad of high-resolution MRI, endoscopy, and digital rectal examination, within a multidisciplinary decision-making framework.

ctDNA demonstrated consistently poor sensitivity for cCR and detection of local regrowth during NOM surveillance, a finding with direct implications for the safety of watch-and-wait protocols. It may be hypothesized that local regrowth frequently arises from deep submucosal niduses with limited vascular access, shedding substantially less DNA into the systemic circulation than haematogenous metastatic deposits. Longitudinal surveillance detected local regrowth in only 40% of patients in the Akiyoshi *et al*.^23^ cohort, and data from Khakoo *et al*.^26^ suggest that only one in ten local regrowth events is preceded by a detectable ctDNA signal during surveillance. Consequently, a watch-and-wait strategy cannot rely on ctDNA surveillance for the detection of minimal residual disease, and the established protocols with MRI, endoscopy, and digital rectal examination must remain the cornerstone of NOM surveillance.

In contrast, post-TNT ctDNA positivity may represent a clinically meaningful signal of systemic disease burden. High specificity and positive predictive value across reporting cohorts indicate that a detectable signal reliably identifies patients at elevated oncological risk, with consistent associations observed across disease-free survival, distant recurrence-free survival, and progression-free survival^12,22,23^. For the multidisciplinary team, persistent post-TNT ctDNA positivity may function as a surrogate marker of chemorefractory disease, identifying a tumour phenotype resistant to standard oxaliplatin-based regimens. In patients who remain ctDNA positive despite an apparently favourable radiological response, a watch-and-wait strategy carries an elevated risk of systemic failure, and these findings should prompt consideration of expedited surgical intervention or enrolment in clinical trials evaluating treatment escalation. Emerging data also suggest that the quantitative tumour fraction may offer greater prognostic resolution than binary ctDNA detection, with a higher post-TNT tumour fraction associated with inferior survival and reduced scCR in the study of Manca *et al*.^22^

Because positive and negative predictive values are influenced by disease prevalence, which was high in these cohorts, likelihood ratios may assist interpretation. Derived from the reported diagnostic data, the negative likelihood ratio was 0.77 in the study of Alden *et al*.^24^ and 0.55 in the study of Akiyoshi *et al*.^23^, consistent with a negative ctDNA result not reliably excluding residual disease.

The generalizability of current evidence is substantially constrained by heterogeneity in assay platforms, detection thresholds, and sampling strategies. The markedly higher post-TNT positivity rate observed in the study of Manca *et al*.^22^ compared with the other included studies most likely reflects differences in assay sensitivity rather than a true biological difference in treatment response. This interpretation is supported by observations in the study of Akiyoshi *et al*.^23^, where high baseline ctDNA positivity was initially attributed to high-risk tumour biology; however, 65.6% of patients were T3a/b and 73.4% were EMVI negative, implicating assay characteristics and detection thresholds as material contributing factors. Detection thresholds were not reported in any included study, precluding direct cross-study comparability and representing a critical gap in the current evidence base. Standardization of assay platforms, detection thresholds, and sampling time points is an essential prerequisite for evidence sufficient to inform clinical practice guidelines. Variation in TNT sequencing across cohorts represents a further source of heterogeneity, although preliminary data from Manca *et al*.^22^ suggest that ctDNA dynamics may not differ significantly between induction and consolidation strategies, providing early evidence that molecular response may be consistent across TNT regimens.

Baseline ctDNA positivity may carry biological significance beyond its prognostic value at restaging. High baseline levels may identify a distinct high-shedding phenotype characterized by aggressive haematogenous dissemination^27^, whereas tumours recurring locally or peritoneally may represent low-shedding phenotypes falling below the detection threshold of current assays, providing a biologically coherent explanation for the consistently poor sensitivity of ctDNA for local regrowth observed across the included studies. This is supported by the significant correlation of post-TNT ctDNA and the presence of EMVI on MRI^23^. This framework remains speculative and requires prospective validation in adequately powered studies.

The interpretive challenges of ctDNA in rectal NOM differ fundamentally from other solid tumour contexts. Unlike stage II/III colon cancer, where post-resection ctDNA unambiguously reflects systemic residual disease, rectal NOM involves a primary tumour that remains *in situ*, creating a complex biological background in which circulating tumour-derived DNA may originate from regressing local disease, occult micrometastases, or both^28^. In addition, ctDNA shedding is volume dependent, and small residual deposits may fall below current detection thresholds regardless of biological activity, further complicating signal interpretation and reinforcing the need for rectal cancer-specific validation frameworks.

ctDNA is one of several approaches studied to supplement anatomical restaging. Patient-derived tumour organoids^29^ and radiomic or deep-learning analysis of restaging MRI^30^ have also been evaluated for response prediction; unlike these tissue- and imaging-based methods, ctDNA reflects systemic rather than local disease, which may contribute to its limited sensitivity for residual local disease.

The practical implementation of ctDNA also requires consideration. Tumour-informed assays require sequencing of the primary tumour and entail a turnaround time and per-patient cost that increase with serial sampling, and ctDNA testing is not currently funded as standard of care within the National Health Service in the UK; routine use would therefore require evidence of incremental clinical value and formal cost-effectiveness evaluation. Standardized sampling time points would additionally allow assessment of ctDNA kinetics in relation to sustained response, and extension to early rectal cancer would require assays of greater analytical sensitivity given the lower disease burden.

This review has several limitations. Heterogeneity in patient selection, TNT sequencing, assay methodology, and clinical response definitions limits cross-study comparability and the interpretability of pooled estimates. NOM-specific subgroups were modest in size, reducing statistical power to detect associations between ctDNA and local regrowth specifically. Apart from the study of Manca *et al*.^22^, the median follow-up time across the included studies was < 3 years, insufficient to capture the full burden of late regrowth and distant metastatic events. The absence of reported detection thresholds across all included studies precludes the definition of clinically actionable cut-off values applicable across platforms. The predominance of studies from high-volume academic centres with access to advanced molecular platforms may limit generalizability to broader clinical practice settings. Each pooled estimate was derived from only three to four studies, with wide confidence intervals, and should be regarded as hypothesis generating. The scCR endpoint combined two distinct entities, sustained cCR without regrowth and pCR after surgery, which likely contributed to its heterogeneity, and several cohorts reported a combined cCR/near-complete response outcome that limits inferences specific to cCR. The proportion of patients achieving cCR or a near-complete response ranged from 26% to 100%, largely reflecting differences in definitions and cohort size. In the two largest cohorts, post-TNT ctDNA was available for only a subset of enrolled patients, specifically 114 of 179 patients in the study of Amatu *et al*.^12^ and in 34 of 251 patients in the study of Yee *et al*.^21^), so the reported associations represent complete-case analyses. Reporting bias was not formally assessed, because fewer than ten studies contributed to each analysis; considered within a GRADE framework, the overall certainty of the evidence was low to very low.

Future studies should prioritize standardized assay protocols, prespecified detection thresholds, and serial sampling frameworks extending through the full surveillance period, with integration of quantitative tumour fraction dynamics to enable more granular individual risk stratification. Until such evidence is established, ctDNA must be interpreted alongside, and not in place of, the anatomical and endoscopic assessment that remains the cornerstone of NOM in locally advanced rectal cancer.

## Supplementary Material

zrag142_Supplementary_Data

## Data Availability

Data are available upon reasonable request from the corresponding author.
