# Supplementary material for: Role of circulating tumour DNA in predicting complete clinical response and local regrowth after total neoadjuvant therapy for rectal cancer: meta-analysis
Source: BJS Open. 2026 Oct 5;10(5):zrag142. doi: 10.1093/bjsopen/zrag142 (PMC13637619; doi:10.1093/bjsopen/zrag142)
Supplement: Supplementary file 1 [file zrag142_supplementary_data.docx]

**Title: The role of circulating tumour DNA in predicting complete clinical response and local regrowth after total neoadjuvant therapy for rectal cancer: systematic review & meta-analysis**

**Nikhil Lal^1,3*^, Siddharth Shukla^2*^, Nicola Hodges^1,3^, Gina Brown^1^, John T Jenkins^1,3^**

^1^ Department of Surgery & Cancer, Imperial College London, London, United Kingdom

^2^ Royal Berkshire NHS Foundation Trust, Reading, United Kingdom

^3^ Complex Cancer Clinic, St Mark's The National Bowel Hospital, London, United Kingdom

**The following authors contributed equally to the manuscript*

**Corresponding author.** *Mr Nikhil Lal, nl516@ic.ac.uk, ORCID: 0000-0002-6889-3496*

**Supplementary Materials – Index**

| Supplementary Table 1. PRISMA 2020 Checklist | pag. 2 |
| --- | --- |
| **Supplementary Methods** | |
| Supplementary Table 2. Search strategy | pag. 6 |
| **Supplementary Results** | |
| Supplementary Table 3. Definitions of clinical response across included studies | pag. 7 |
| Supplementary Table 4. Timing of ctDNA sampling across treatment phases | pag. 7 |
| Supplementary Table 5. ctDNA assay characteristics across included studies | pag. 8 |
| Supplementary Table 6. Risk of bias assessment (ROBINS-I) | pag. 9 |
| Supplementary Table 7. GRADE certainty-of-evidence assessment | pag. 10 |
| **Supplementary Appendixes** | |
| Not applicable | pag. 11 |
| **Supplementary Figures and Tables** | |
| Not applicable — supplementary tables are presented within the sections above | pag. 11 |

**Supplementary Table 1: PRISMA 2020 Checklist**

| **Section and Topic** | **Item #** | **Checklist item** | **Location where item is reported** |
| --- | --- | --- | --- |
| **TITLE** | | | |
| Title | 1 | Identify the report as a systematic review. | p.1, lines 1–3 |
| **ABSTRACT** | | | |
| Abstract | 2 | See the PRISMA 2020 for Abstracts checklist. | p.2, lines 29–86 (structured abstract; see PRISMA 2020 for Abstracts checklist) |
| **INTRODUCTION** | | | |
| Rationale | 3 | Describe the rationale for the review in the context of existing knowledge. | p.4–5, lines 65–124 (Introduction) |
| Objectives | 4 | Provide an explicit statement of the objective(s) or question(s) the review addresses. | p.5, lines 119–124 |
| **METHODS** | | | |
| Eligibility criteria | 5 | Specify the inclusion and exclusion criteria for the review and how studies were grouped for the syntheses. | p.6, lines 138–142 (eligibility criteria); grouping for synthesis p.8, line 182 |
| Information sources | 6 | Specify all databases, registers, websites, organisations, reference lists and other sources searched or consulted to identify studies. Specify the date when each source was last searched or consulted. | p.6, lines 132–136 (databases searched, search date); full listing in Table S2, p.23, line 665 |
| Search strategy | 7 | Present the full search strategies for all databases, registers and websites, including any filters and limits used. | Table S2 (Search Strategy), p.23, line 665; referenced in-text p.6, line 136 |
| Selection process | 8 | Specify the methods used to decide whether a study met the inclusion criteria of the review, including how many reviewers screened each record and each report retrieved, whether they worked independently, and if applicable, details of automation tools used in the process. | p.6, lines 147–150 (two reviewers, NL and SS, independent screening with third-reviewer arbitration; no automation tools used) |
| Data collection process | 9 | Specify the methods used to collect data from reports, including how many reviewers collected data from each report, whether they worked independently, any processes for obtaining or confirming data from study investigators, and if applicable, details of automation tools used in the process. | p.6–7, lines 151–159 list the variables extracted, but the number of reviewers performing data extraction and whether independently is not explicitly stated — recommend a brief addition |
| Data items | 10a | List and define all outcomes for which data were sought. Specify whether all results that were compatible with each outcome domain in each study were sought (e.g. for all measures, time points, analyses), and if not, the methods used to decide which results to collect. | p.7, lines 156–165 (three prespecified outcomes: cCR, scCR, survival) |
|  | 10b | List and define all other variables for which data were sought (e.g. participant and intervention characteristics, funding sources). Describe any assumptions made about any missing or unclear information. | p.7, lines 152–156 |
| Study risk of bias assessment | 11 | Specify the methods used to assess risk of bias in the included studies, including details of the tool(s) used, how many reviewers assessed each study and whether they worked independently, and if applicable, details of automation tools used in the process. | p.7, lines 170–178 |
| Effect measures | 12 | Specify for each outcome the effect measure(s) (e.g. risk ratio, mean difference) used in the synthesis or presentation of results. | p.2, line 42 (Abstract); p.8, line 185 (Methods) |
| Synthesis methods | 13a | Describe the processes used to decide which studies were eligible for each synthesis (e.g. tabulating the study intervention characteristics and comparing against the planned groups for each synthesis (item #5)). | p.8, line 182 |
|  | 13b | Describe any methods required to prepare the data for presentation or synthesis, such as handling of missing summary statistics, or data conversions. | p.8, line 184 (construction of 2×2 contingency tables) |
|  | 13c | Describe any methods used to tabulate or visually display results of individual studies and syntheses. | Figures 2–4, p.22, lines 634–652; Tables 1–2, p.23, lines 654–656 |
|  | 13d | Describe any methods used to synthesize results and provide a rationale for the choice(s). If meta-analysis was performed, describe the model(s), method(s) to identify the presence and extent of statistical heterogeneity, and software package(s) used. | p.8, lines 185–191 |
|  | 13e | Describe any methods used to explore possible causes of heterogeneity among study results (e.g. subgroup analysis, meta-regression). | p.8, line 191 (subgroup analyses not performed owing to the limited number of eligible studies) |
|  | 13f | Describe any sensitivity analyses conducted to assess robustness of the synthesized results. | Not reported — no sensitivity analyses were described; recommend an explicit statement |
| Reporting bias assessment | 14 | Describe any methods used to assess risk of bias due to missing results in a synthesis (arising from reporting biases). | Not addressed as a separate methods statement; formal reporting-bias assessment was not performed as fewer than ten studies contributed to each analysis, stated in Limitations, p.17, lines 450–452 |
| Certainty assessment | 15 | Describe any methods used to assess certainty (or confidence) in the body of evidence for an outcome. | p.7, lines 178–180 |
| **RESULTS** | | | |
| Study selection | 16a | Describe the results of the search and selection process, from the number of records identified in the search to the number of studies included in the review, ideally using a flow diagram. | p.9, lines 208–217; Figure 1, p.22, line 632 |
|  | 16b | Cite studies that might appear to meet the inclusion criteria, but which were excluded, and explain why they were excluded. | Figure 1 (p.22, line 632) reports aggregate full-text exclusion counts by reason (n=28: not TNT-specific n=4, reporting only pCR n=6, not LARC-specific n=10, insufficient outcome data n=8); individual excluded studies are not cited by name |
| Study characteristics | 17 | Cite each included study and present its characteristics. | p.9, lines 217–221; Table 1, p.23, line 654 |
| Risk of bias in studies | 18 | Present assessments of risk of bias for each included study. | p.12, lines 299–301; Supplementary Table 6, p.23, line 673 |
| Results of individual studies | 19 | For all outcomes, present, for each study: (a) summary statistics for each group (where appropriate) and (b) an effect estimate and its precision (e.g. confidence/credible interval), ideally using structured tables or plots. | Table 2, p.23, line 656; narrative results p.12–13, lines 278–298 |
| Results of syntheses | 20a | For each synthesis, briefly summarise the characteristics and risk of bias among contributing studies. | p.10–11, lines 262–274 |
|  | 20b | Present results of all statistical syntheses conducted. If meta-analysis was done, present for each the summary estimate and its precision (e.g. confidence/credible interval) and measures of statistical heterogeneity. If comparing groups, describe the direction of the effect. | p.11, lines 266 (OR 8·01, 95% CI 1·43–44·90) and 274 (I²=59·9%); Figures 3–4, p.22, lines 638–652 |
|  | 20c | Present results of all investigations of possible causes of heterogeneity among study results. | Not applicable — no subgroup analysis or meta-regression was performed (p.8, line 191) |
|  | 20d | Present results of all sensitivity analyses conducted to assess the robustness of the synthesized results. | Not reported — no sensitivity analyses were described |
| Reporting biases | 21 | Present assessments of risk of bias due to missing results (arising from reporting biases) for each synthesis assessed. | Not presented as a separate Results subsection; stated in Limitations, p.17, lines 450–452, that formal reporting-bias assessment was not performed because fewer than ten studies contributed to each analysis |
| Certainty of evidence | 22 | Present assessments of certainty (or confidence) in the body of evidence for each outcome assessed. | p.12, line 304; Supplementary Table 7, p.23, line 676 |
| **DISCUSSION** | | | |
| Discussion | 23a | Provide a general interpretation of the results in the context of other evidence. | p.13, lines 316–354 |
|  | 23b | Discuss any limitations of the evidence included in the review. | p.16–17, lines 431–452 |
|  | 23c | Discuss any limitations of the review processes used. | p.16–17, lines 431–452 |
|  | 23d | Discuss implications of the results for practice, policy, and future research. | p.14–16, lines 358–426 |
| **OTHER INFORMATION** | | | |
| Registration and protocol | 24a | Provide registration information for the review, including register name and registration number, or state that the review was not registered. | p.6, lines 129–130 (PROSPERO CRD420261322560) |
|  | 24b | Indicate where the review protocol can be accessed, or state that a protocol was not prepared. | p.6, lines 129–130 (PROSPERO registration record serves as the protocol) |
|  | 24c | Describe and explain any amendments to information provided at registration or in the protocol. | Not reported — no amendments are described; consider noting the post-hoc addition of the GRADE certainty assessment as an amendment |
| Support | 25 | Describe sources of financial or non-financial support for the review, and the role of the funders or sponsors in the review. | p.1, line 15; p.21, line 612 |
| Competing interests | 26 | Declare any competing interests of review authors. | p.21, line 610 |
| Availability of data, code and other materials | 27 | Report which of the following are publicly available and where they can be found: template data collection forms; data extracted from included studies; data used for all analyses; analytic code; any other materials used in the review. | p.1, line 22 (data available upon request) |

*From: Page MJ, McKenzie JE, Bossuyt PM, Boutron I, Hoffmann TC, Mulrow CD, et al. The PRISMA 2020 statement: an updated guideline for reporting systematic reviews. BMJ 2021;372:n71. This work is licensed under CC BY 4.0.*

**Supplementary Table 2**

| **Database** | **Search Strategy** |
| --- | --- |
| **PubMed (MEDLINE)** | ("circulating tumour DNA" OR "circulating tumor DNA" OR ctDNA OR "cell-free DNA" OR cfDNA) AND ("rectal cancer" OR "rectal adenocarcinoma" OR "rectal neoplasm" OR "locally advanced rectal cancer" OR LARC) AND ("total neoadjuvant therapy" OR TNT OR "neoadjuvant chemoradiotherapy" OR chemoradiation) AND ("clinical complete response" OR cCR OR "pathological complete response" OR pCR OR "non-operative management" OR "watch and wait" OR recurrence OR "minimal residual disease") |
| **EMBASE** | ('circulating tumor dna'/exp OR ctDNA OR 'cell free dna'/exp OR cfDNA) AND ('rectum cancer'/exp OR 'rectal adenocarcinoma' OR 'locally advanced rectal cancer' OR LARC) AND ('neoadjuvant therapy'/exp OR 'total neoadjuvant therapy' OR TNT OR 'chemoradiotherapy'/exp) AND ('clinical complete response' OR cCR OR 'pathological complete response' OR pCR OR 'non operative management' OR 'watch and wait' OR recurrence OR 'minimal residual disease') |
| **Web of Science** | TS=(ctDNA OR "circulating tumour DNA" OR "cell-free DNA") AND TS=("rectal cancer" OR "rectal adenocarcinoma" OR "locally advanced rectal cancer") AND TS=("total neoadjuvant therapy" OR TNT OR chemoradiotherapy) AND TS=("clinical complete response" OR cCR OR "watch and wait" OR recurrence OR "minimal residual disease") |

**Supplementary Table 2: Search Strategy**

**Supplementary Results**

**Supplementary Table 3**

| **Study** | **Definition of clinical response** |
| --- | --- |
| **Kim et al.** | cCR defined as no residual tumour on DRE, a white scar and/or telangiectasia on endoscopy, and no intermediate T2 signal or diffusion restriction on MRI |
| **Yee et al.** | Sustained CR defined as clinical CR without local regrowth for ≥ 1 year, or pathological CR (TRG 0) in patients undergoing surgery within 1 year |
| **Amatu et al.** | cCR defined by multimodal assessment using endoscopy, DRE, and MRI at post-TNT restaging |
| **Manca et al.** | Sustained clinical complete response (scCR) defined using OPRA criteria (endoscopy and DRE), incorporating absence of regrowth over follow-up |
| **Akiyoshi et al.** | Composite clinical response including cCR and near-complete response (nCR) based on clinical and endoscopic assessment at restaging |
| **Alden et al.** | cCR defined by absence of residual tumour on multimodal assessment (DRE, endoscopy, and MRI) following TNT |

**Supplementary Table 3. Definitions of clinical response across included studies.**

**Supplementary Table 4**

| **Study** | **Baseline** | **During TNT** | **Post-TNT (Restaging)** | **Surveillance** |
| --- | --- | --- | --- | --- |
| **Kim 2025** | Pre-TNT | Not performed | NR | Serial follow-up (interval not specified) |
| **Alden 2024** | Pre-TNT | During TNT (interval not specified) | Median ~1 week post-TNT | Serial follow-up (interval not specified) |
| **Akiyoshi 2025** | Pre-TNT | During TNT (sequential sampling) | NR | Longitudinal surveillance (serial) |
| **Manca 2025** | Pre-TNT | During TNT (early on-treatment) | Median 8 weeks post-TNT | Intensive longitudinal follow-up |
| **Yee 2025** | Pre-TNT | Not performed | Within 90 days post-TNT | Not reported |
| **Amatu 2025** | Pre-TNT | Not performed | 11–17 weeks post-TNT | Serial follow-up (interval not specified) |

**Supplementary Table 4. Timing of ctDNA sampling across treatment phases.**

**Supplementary Table 5**

| **Study** | **Assay Type** | **Platform** | **Panel / Target** | **Detection Threshold** |
| --- | --- | --- | --- | --- |
| **Akiyoshi et al.** | Tumour-informed | Signatera | Personalised panel | NR |
| **Alden et al.** | Tumour-informed | Signatera | Personalised panel | NR |
| **Amatu et al.** | Tumour-agnostic | Guardant Reveal | Epigenomic assay | NR |
| **Kim et al.** | Tumour-informed | Signatera | Patient-specific SNVs | NR |
| **Yee et al.** | Mixed | Signatera, Guardant, Tempus | Commercial panels | NR |
| **Manca et al.** | Tumour-informed | C2i Genomics | Whole-genome (tumour fraction) | NR |

**Supplementary Table 5. ctDNA assay characteristics across included studies.**

**Supplementary Table 6**

| **Study** | **Confounding** | **Selection** | **Exposure (ctDNA)** | **Deviations** | **Missing data** | **Outcome measurement** | **Reporting bias** | **Overall risk** |
| --- | --- | --- | --- | --- | --- | --- | --- | --- |
| **Alessio Amatu (2025)** | H | L | L | L | L | L | L | L |
| **Jared T. Yee (2025)** | H | L | L | L | L | L | L | M |
| **Jin K. Kim (2025)** | H | L | L | L | L | M | L | M |
| **Stephanie Alden (2024)** | M | L | L | L | L | L | L | M |
| **Paolo Manca (2025)** | L | L | L | L | L | L | L | L |
| **Takashi Akiyoshi (2025)** | M | L | L | L | L | L | L | L |

**Supplementary Table 6: Risk of bias assessed using a modified ROBINS-I framework. L = low risk; M = moderate risk; H = high risk.**

**Supplementary Table 7**

| **Outcome** | **No. of studies (participants)** | **Study design** | **GRADE assessment** | **Certainty** |
| --- | --- | --- | --- | --- |
| **ctDNA negativity and cCR** | 4 (250) | Observational | No serious concerns beyond observational design; consistency acceptable (I²=27%); pooled OR 8·01 (95% CI 1·43–44·90) excludes no effect | Low |
| **ctDNA status and sustained cCR** | 3 (111) | Observational | Downgraded for serious inconsistency (I²=59·9%) and serious imprecision (95% CI 0·03–394·64); composite endpoint | Very low |
| **Post-TNT ctDNA positivity and survival (DFS/DRFS/PFS)** | 4 (narrative) | Observational | Consistent direction of effect across cohorts (e.g. DFS HR 6·7, Akiyoshi; DRFS HR 0·38, Amatu; DFS HR 4·20, Manca); not pooled | Low |
| **Post-TNT ctDNA for residual disease or local regrowth (diagnostic accuracy)** | 3 (narrative) | Observational | Downgraded for serious inconsistency (sensitivity 23–76·5%) and serious imprecision (small event numbers) | Very low |

**Supplementary Table 7: GRADE certainty-of-evidence assessment for pooled and narratively synthesised outcomes. Observational evidence begins at low certainty under GRADE. cCR, clinical complete response; CI, confidence interval; DFS, disease-free survival; DRFS, distant recurrence-free survival; HR, hazard ratio; OR, odds ratio; PFS, progression-free survival.**

**Supplementary Appendixes**

*Not applicable.*

**Supplementary Figures and Tables**

*Not applicable. All supplementary tables for this review (Supplementary Tables 1–7) are presented within the sections above.*
